# Treatment of Tuna Cooking Juice via Ceramic Ultrafiltration Membrane: Optimization Using Response Surface Methodology

**DOI:** 10.3390/membranes12080813

**Published:** 2022-08-22

**Authors:** Wala Aloulou, Hajer Aloulou, Afef Attia, Sudip Chakraborty, Raja Ben Amar

**Affiliations:** 1 Research Unit ‘Advanced Technologies for Environment and Smart Cities’, Faculty of Science of Sfax, University of Sfax, Sfax 3038, Tunisia; 2 Department of Computer Engineering, Modeling, Electronics and Systems (D.I.M.E.S.), University of Calabria, Via-P. Bucci, Cubo-42A, 87036 Rende, Italy

**Keywords:** ultrafiltration, tuna cooking juice, response surface methodology, concentration polarization

## Abstract

In the present work, optimized ultrafiltration conditions, using a ceramic multi tubular titania membrane (150 KDa), were investigated for the treatment of tuna cooking juice, for water reuse in the industrial process. The interactive effects of the volume concentrating factor (VCF) (1.03–4.25), feed temperature (T) (20–60 °C), and applied transmembrane pressure (ΔP) (2–5 bar) on protein removal (R protein) and permeate flux (J) were determined. A Box–Behnken experimental design (BBD) with the response surface methodology (RSM) was used for statistical analysis, modeling, and optimization of the operating conditions. The analysis of variance (ANOVA) results proved that the protein removal and permeate flux were significant and represented good correlation coefficients of 0.9859 and 0.9294, respectively. Mathematical modeling showed that the best conditions were VCF = 1.5 and a feed temperature of 60 °C, under a transmembrane pressure of 5 bar. The fouling mechanism was checked by applying a polarization concentration model. Determination of the gel concentration confirmed the results found in the mass balance calculation and proved that the VCF must not exceed 1.5. The membrane regeneration efficiency was proven by determining the water permeability after the chemical cleaning process.

## 1. Introduction

Today, the consumption of seafood has significantly increased, due to its richness in protein with high quality, and the presence of certain nutrients with higher added value benefit to the consumer’s health. The increase of marine product consumption leads to an increase in the seafood industry’s activities. Consequently, large quantities of by-products generated by these industries are discharged as waste, such as protein, head viscera, and bones, without regenerating or valorizing [1]. In particular, in Tunisia, tuna is the largest commercial canned fishery product. Tuna cooking juice presents high organic and salt contents, which necessitate its treatment before release into the environment. As cooking juices are rich in aromas and proteins of interest in the food or feed sectors, processes that reduce pollution load and recover valuable compounds are worth investigating [2].

Currently, soluble proteins are recovered via chemical methods such as pH shifting [3,4], three-phase partitioning [5], or two-phase aqueous extraction [6]. Unfortunately, these techniques apply many chemicals and solvents. They can lead to the extraction of low-purity proteins with high salt content, denaturation, and poor functional properties, or can be incompatible with industrial-scale applications. In this context, membrane filtration technologies, especially ultrafiltration, are presented as advantageous methods to purify and concentrate components of microalgal biomass under mild operating and chemical-free conditions [7,8,9]. Membrane filtration is notably helpful for the concentration of large volumes and can be easily automatized and scaled up to an industrial level [10]. In addition, applying a membrane separation process can reduce the denaturation, deactivation, and degradation of biological molecules of interest [11].

The response surfaces methodology (RSM) seems to be a prevalent method and one that needs optimization. There are several other modeling approaches, whereas RSM is a numerical approach to multifactorial analysis of experimental design and process optimization [12]. This methodology offers a better comprehension of the process than the standard experimental methods, since it can calculate how the inputs affect the outputs in a complex process involving the interaction between factors [13]. RSM is performed in three steps: the first is the analysis of individual and combined parameters. In the second step, the influence of the primary variables is studied for process effectiveness. The third is the process optimization using a RSM-based regression model to determine the optimized conditions [14]. In particular, RSM based on BBD is generally utilized for its numerous advantages, such as the lower number of required experiments compared to a three-level full factorial design. Simultaneously, it is more successful than central composite designs (CCD) [15,16].

The main goal of this work was to investigate the possibility of ultrafiltration of tuna cooking juice for maximum protein recovery and, at the same time, water purification with high performances under different conditions of VCF, (T), and (ΔP). Protein removal and stabilized permeate flux were then determined. Statistical data analysis was carried out to obtain a suitable mathematical model for the process. Finally, the model was applied, and the influence of the different factors on the protein retention and stabilized permeate flux were discussed [17].

## 2. Materials and Methods

### 2.1. Tuna Cooking Juices Collection

Tuna cooking juices were collected from a Seafood industry (BK-FOOD) in Sid El Heni Sousse, Tunisia. The characteristics of three different collected effluents are summarized in Table 1. At first, the wastewater was pre-filtered using a porous filter paper of 60 μm to remove free solid particles that could clog the membranes.

### 2.2. Ultrafiltration Process

**Figure 1:**
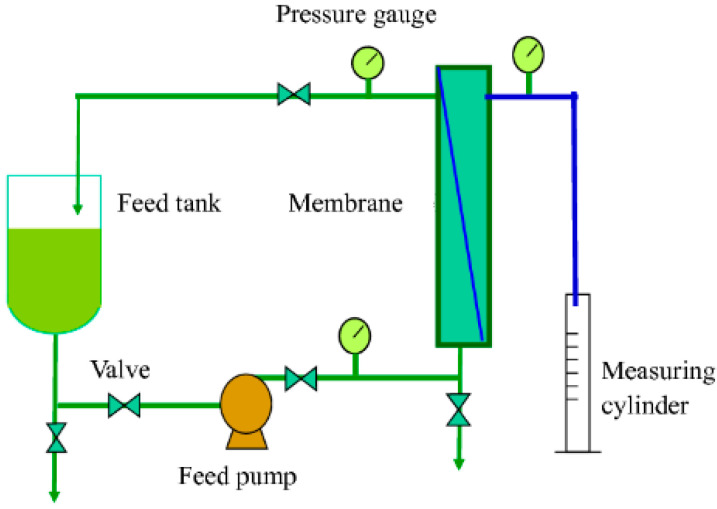
Schematic representation of the cross-flow ultrafiltration experiment set-up.

The cross-flow ultrafiltration experiments were performed using a semi-pilot scale set-up, presented schematically in Figure 1. The installation was equipped with automated systems for controlling the feed flow rate and temperature. The module with a tubular ceramic membrane was mounted in the installation. A UF titania multi-channel (7 channels) membrane with an area of 0.155 m^2^, separation cut-off 150 KDa, and water permeability of 230 L/h·m^2^.bar was tested in this study under a transmembrane pressure in the range of 2–5 bar, and temperature range from 20 to 60 °C. 

The evolution of the permeate flux was measured in the course of the concentration of the solution. The permeate flux was calculated using the following equation [18]:

$$J=\frac{V}{A\cdot t}$$

where *J* is the permeate flux (L/m^2^·h), *V* the volume of permeate (L), *A* the membrane surface (m^2^), and *t* the time of ultrafiltration (h).

The volume concentrating factor (*VCF*) was calculated as:

(2) $$VCF=\frac{V_{i}}{V_{C}}$$

*V_i_* and *V_c_* are, respectively, the initial and retentate volumes.

### 2.3. Analytical Methods 

The conductivity and pH were measured using a conductivity meter (EC-400L, Istek, Seoul, Korea) and a pH meter (pH-220L, Istek, Korea). The turbidity was measured by a turbidimeter (model 2100A, Hach, Düsseldorf, Germany) in agreement with standard method 2130B. This study used the Lowry method (Bio-Rad DC Protein Assay; cat no. 500-0116) for protein concentration.

For the evaluation of UF rejection, the percentage reduction of different parameters (protein and turbidity) was determined as follows [19,20]:

(3) $$R(\%)=\frac{\left(C_{f}-C_{p}\right)}{C_{f}}\times 100$$

where *C_f_* and *C_p_* represent the concentration of pollutants in the feed and in the permeate, respectively.

### 2.4. Experimental Design Methodology 

The response surface methodology (RSM) was applied to evaluate the effects of ultrafiltration parameters and optimize various conditions for different responses. Table 2 summarizes the studied variables: volume concentrating factor (X_1_), temperature (X_2_), and transmembrane pressure (X_3_). A Box–Behnken experimental design (BBD) was studied using three numeric factors on three levels [20]. The BBD included 13 randomized runs with one replicate at the central point.

The matrix, experimental range, and responses are presented in Table 3. 

RSM is a statistical method of multifactorial analysis of experimental data, which allows a higher understanding of the process compared to the standard methods of experimentation, due to the ability to predict how the inputs affect the outputs in a complex process where different factors can interact among themselves. All the coefficients of the different polynomial equations were tested for significance with an analysis of variance (ANOVA) [21]. For responses obtained after the experiments (retention of protein and permeate flux), a polynomial model of the second degree was established to evaluate and quantify the influence of the variables:

(4) $$Y(\%)=b_{0}+\sum b_{i}X_{i}+\sum \sum b_{ij}X_{i}X_{j}+\varepsilon ;\;\;i\neq j$$

where *X_i_* and *X_j_* are the coded variables (−1 or +1), *b*_0_ the mean of the responses obtained, *b_i_* the main effect of factor *i* for the response *Y*, *b_ij_* the interaction effect between factors *i,* and *j* for the response and represents the error on the response.

(5) $$Y(\%)=b_{0}+\sum b_{i}X_{i}+\sum \sum b_{ij}X_{i}X_{j}+\sum \sum b_{ii}X_{i}{}^{2}+\varepsilon ;\;\;i\neq j$$

where *Y*, *b*_0_, *b_i_*, *b_ii_*, *b_ij_*, *X_i_*, and *X_j_* represent the predicted response, the constant coefficient, the linear coefficient, the interaction coefficient, the quadratic coefficient, and the coded values of the factors, respectively. The sufficiency of the model was evaluated using the coefficient of determination (R^2^) and model *p*-value. Statistical analysis was evaluated using Statistica software. Response surface plots are indicated for two factors, where the third factor is set to its medium value.

### 2.5. Investigation of Fouling Mechanism

**Figure 2:**
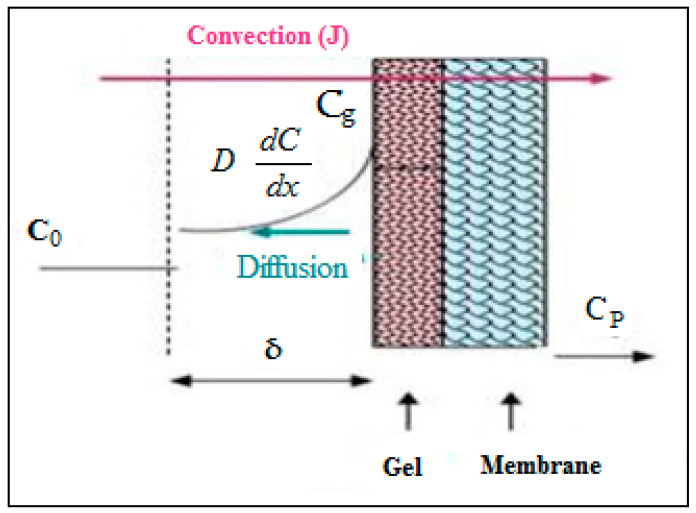
Schematic representation of concentration polarization phenomena.

To determine the fouling mechanism that occurred during the UF of tuna cooking juice, a mathematical model established for polarization concentration phenomena was applied [22,23]. In 1970, a classic paper by Michaels and coworkers [24] provided the first comprehensive analysis of concentration polarization in UF and introduced the term “gel polarization”. The limiting resistance to permeate flux is in the dynamically formed secondary or gel layer. It is possible to calculate the water flux through the membrane based on the mass transfer of retained species (dissolved solutes or colloidal materials) from the membrane surface back into the bulk stream [25] (Figure 2). The dynamic gel layer is assumed to have a fixed gel concentration (*C_g_*) but is free to vary in thickness or porosity (varying permeability or resistance to flow). In addition, the solvent flux (*J_v_*) will be independent of the pressure-driving force or the membrane permeability. This is due to the gel layer’s resistance to flux, which will adjust until the convective transport of retained species to the membrane surface (*J_v_C*). The solvent is equal to the back-diffusive transport (*D*(d*C*/d*x*) (Figure 2).

Thus, at a steady state:

(6) $$J_{v}=-D\frac{dC}{dx}$$

where *D* is the diffusion coefficient for solute transport through the solvent, *C* is the concentration of membrane-retained solutes or colloidal species, and *dC*/*dx* is the solute concentration gradient. The gel-polarization model (Equation (6)) can be integrated since the boundary conditions are specified; the solute concentration at the membrane surface is fixed at an upper limit (i.e., saturation *C_g_*), and the bulk-stream concentration is known (*C_b_*). Therefore, where the thickness of the boundary layer over which the concentration of the solute varies (Equation (7)):

(7) $$J_{v}=\frac{D}{\delta }Ln(\frac{C_{g}}{C_{b}})$$

It is assumed that under conditions where the gel polarization model holds, the flux through the membrane is invariant with a transmembrane pressure drop or permeability. This depends only on the solute characteristics (*D* and *C_g_*) and the boundary layer thickness. A weakness is that this model cannot describe the whole range of flux–pressure dependency. Fluid management techniques must be directed towards decreasing the boundary layer thickness or, put another way, towards increasing the mass transfer coefficient, *k* where:

(8) $$J_{v}=kLn(\frac{C_{g}}{C_{b}})$$

(9) $$k=\frac{D}{\delta }$$

The validity of Equations (8) and (9) has been demonstrated for a large number of macromolecular solutes and colloidal species. The well-known mass transfer–heat transfer analogies in the chemical engineering literature evaluate the possible mass transfer coefficient, *k*, and provide insight into how membrane geometry and fluid flow conditions can be specified to optimize the flux.

### 2.6. Fouling Resistance Abilities and Membrane Regeneration 

The membrane fouling resistance ability of the used UF ceramic membrane (150 KDa) was evaluated at concentration initial of protein = 7.28 g/L, T = 60 °C, and ΔP = 5 bar by the determination after one hour of filtration. Four parameters, namely flux decay ratio (*FDR*), flux recovery ratio (*FRR*), reversible flux decline ratio (*RFR*), and irreversible flux decline ratio (*IFR*) can be calculated as in the following equations [26,27]:

(10) $$FDR=\frac{J_{w}-J_{s}}{J_{w}}\times 100$$

(11) $$FRR=\frac{J_{w}{}_{a}}{J_{w}}\times 100$$

(12) $$RFR=\frac{J_{w}-J_{s}}{J_{ws}}\times 100$$

(13) $$IFR=\frac{J_{w}-J_{wa}}{J_{w}}\times 100$$

*J_w_* is the water flux of the new membrane, and *J_S_* is the stabilized permeate flux during the UF using cooking juice tuna wastewater. *J_wa_* is the membrane’s water permeate flux measured after cleaning the membrane with distilled water after wastewater purification. Membrane regeneration was accomplished, first by rinsing with distilled water, then by using an acid-base treatment with an alternative circulation of 2% solutions of NaOH at 80 °C and HNO_3_ at 60 °C for 30 min. Finally, the membrane was washed with distilled water until at neutral pH. The efficacy of the cleaning protocol was cheeked by measuring the initial water permeability after the cleaning cycle.

## 3. Results and Discussion

### 3.1. Wastewater UF

UF of industrial tuna cooking juice collection using a titania ceramic membrane (150 KDa) was efficient concerning the obtained stabilized permeate flux and the retention of different parameters (protein, turbidity, and permeate flux). It is worth noting that the UF process achieved almost total turbidity retention, regardless of the initial values and the treatment conditions. The protein removal and permeate flux results show that they were affected by different parameters, such as the VCF, (T), and (ΔP).

### 3.2. Protein Removal Response

Table 4 illustrates the regression coefficients obtained by ANOVA for the quadratic model for protein removal and the modified quadratic model for permeate flux. The *p*-value determined the significance of input factors and their interactions in the studied model. A factor affects the response if the *p*-value is less than the used probability level. The significance was judged at probability levels less than 0.05 [28].

From the results (Table 4), it was found that the linear model terms and the quadratic model of VCF X_1_, X_11_ were significant (*p*-value < 0.05), indicating that only this independent variable had a distinct effect on the protein removal. The coefficient R^2^ = 0.9859 showed that the model fit was significant, according to Joglekar et al. [29], which proved that the model fit was good when R^2^ > 0.80.

Furthermore, R^2^ evaluates the discrepancy or variance in the apparent values, which could be explained by the independent variables and their interactions over the design of the specific factors. The value of R^2^ = 0.9859 shows that the model could describe the response variation of 98.59% of the total variation, and only 1.41% of it could not be explained by the empirical model. 

**Figure 3:**
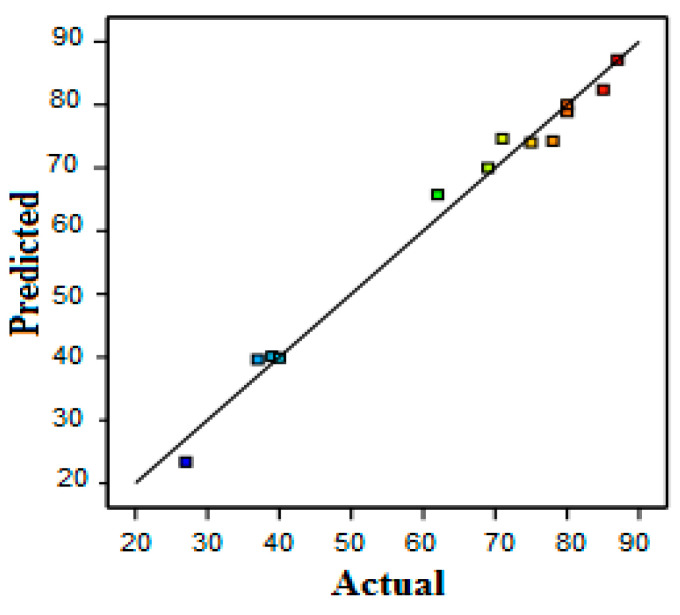
Comparison of calculated and predicted values for protein removal using RSM. The difference of color for the dots is due to the difference between min and max values of the parameter: min in blue and max in red (given by the software).

A comparison of the experimental results (actual values) and the values predicted by the model is presented in Figure 3.

**Figure 4:**
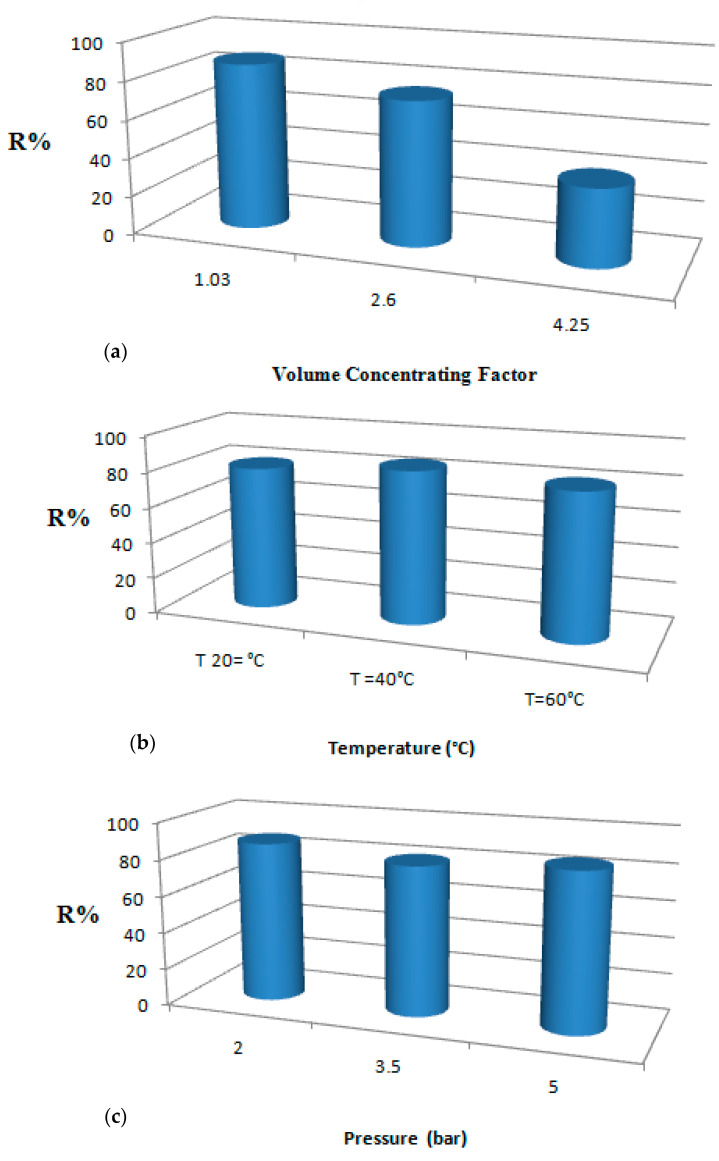
Variation of protein retention with VCF (**a**), temperature (**b**), and pressure (**c**).

The theoretical and experimental values are very close for protein removal. This proximity reflects the robustness of the statistical models obtained. In addition, the experimental results proved that the protein removal was only affected by the VCF (Figure 4).

### 3.3. Permeate Flux Response

**Figure 5:**
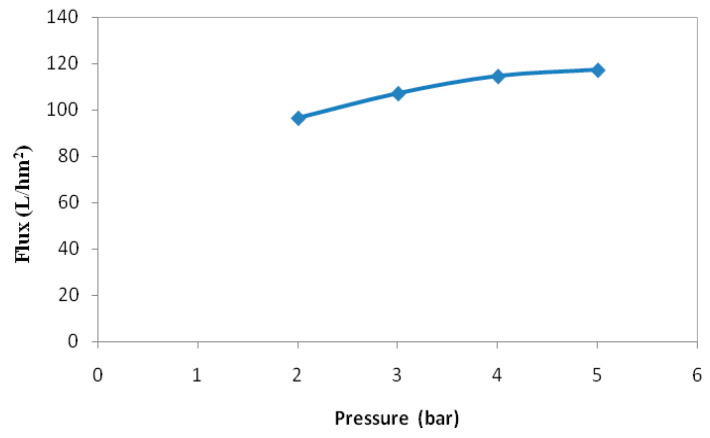
Evolution of stabilized permeate flux with applied pressure at: VCF = 1.03 g/L, and T = 20 °C.

The results of the effect of input factors on permeate flux values are given and analyzed. The modified quadratic model proved that the linear model terms of VCF (X_1_) and temperature (X_2_) were significant (*p*-value < 0.05), but the applied transmembrane pressure did not affect the permeate flux. This estimated result correlates with the experimental results, showing that the stabilized permeate flux was almost stable, around 110 L/h·m^2^ for a pressure of 3 bar under the experimental conditions: VCF = 1.03 and T = 20 °C (Figure 5).

**Figure 6:**
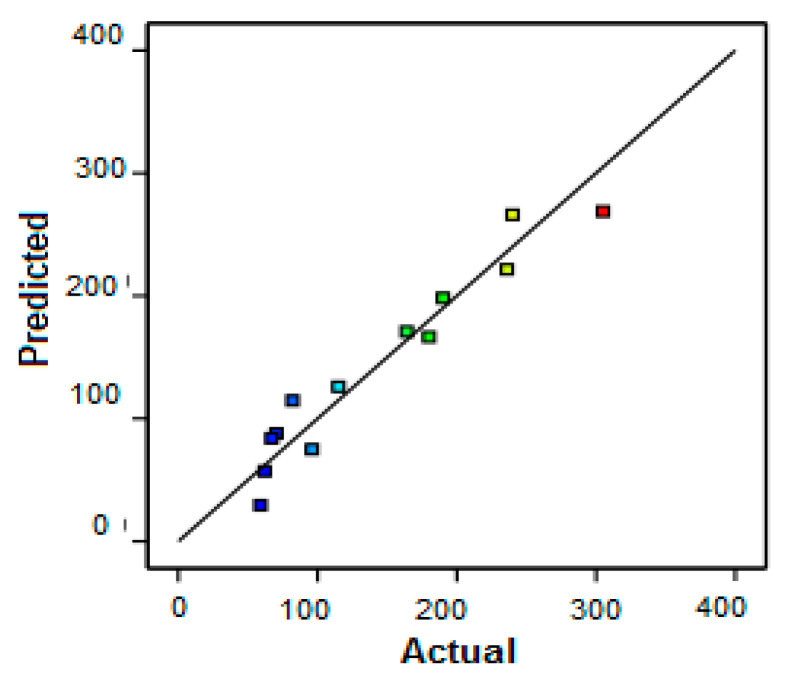
Comparison of calculated and predicted values for permeate flux by RSM. The difference of color for the dots is due to the difference between min and max values of the parameter: min in blue and max in red (given by the software).

The value of R^2^ = 0.9294 confirms that the model fit is significant. Additionally, the model allowed determining the variability in the observed response values, which can be described by the independent factors and their interactions over the range of the corresponding factors. The value of R^2^ = 0.9294 indicates that the model could describe 92.94% of the total variation, and the model could not describe only 7.06% of it. Furthermore, Figure 6 suggests that the experimental results of permeate flux value were close to the predicted values.

### 3.4. Optimization of Permeate Flux and Protein Removal 

**Figure 7:**
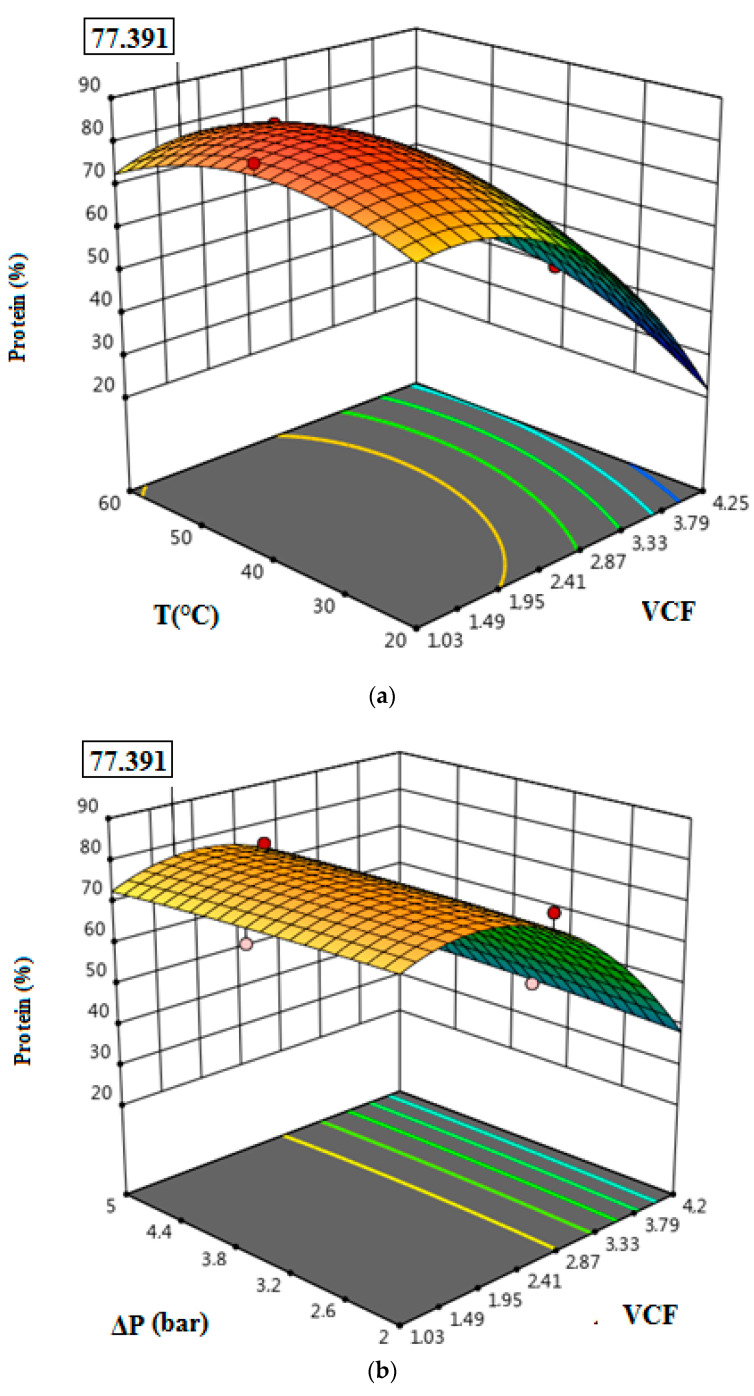

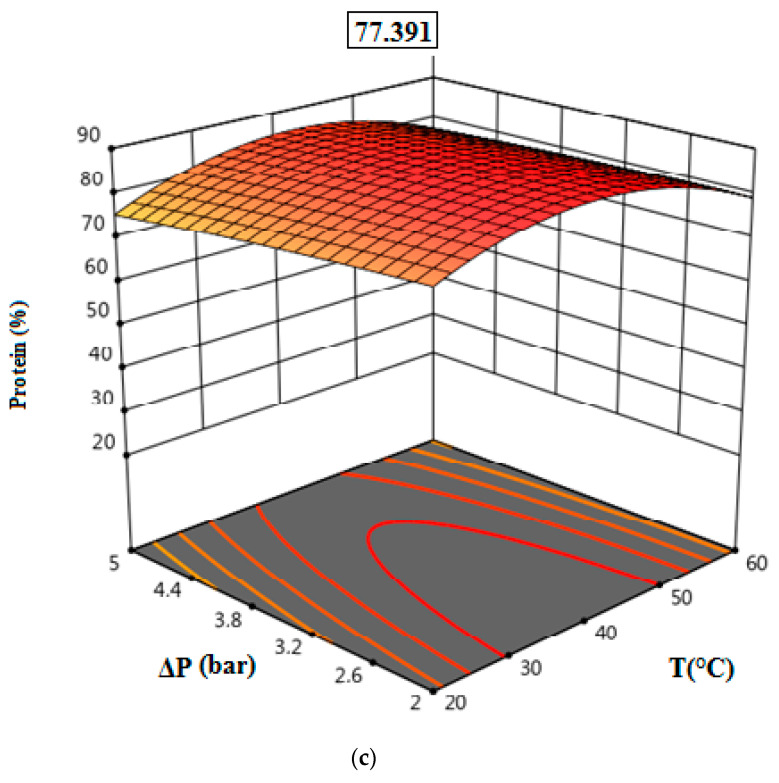
Response surface plots showing the effects of variables on protein removal: The interaction of VCF and T (**a**); the interaction of VCF and ΔP (**b**); the interaction of T and ΔP (**c**).

**Figure 8:**
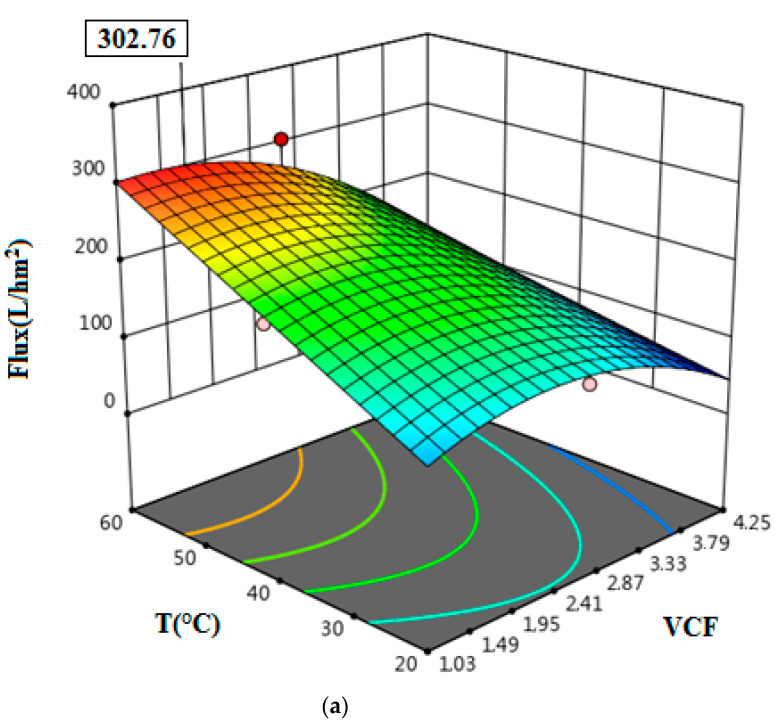

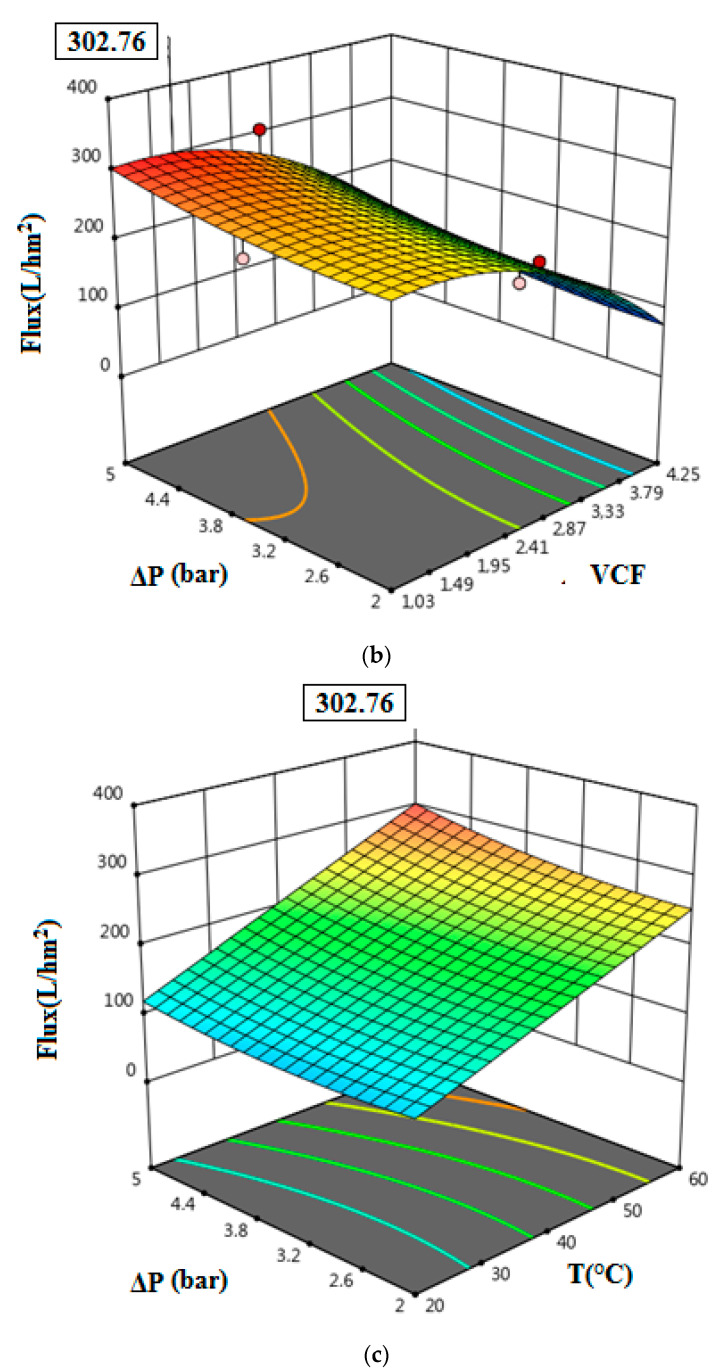
The response surface plots showing the effects of variables on permeate flux: The interaction of VCF and T (**a**); the interaction of VCF and ΔP (**b**); the interaction of T and ΔP (**c**).

The optimizations using RSM were performed by maximizing the protein removal and permeate flux. In Figure 7a–c and Figure 8a–c, the responses’ three-dimensional surfaces, obtained with the proposed quadratic degree model, can be observed. The interaction of independent variables in the treatment of tuna cooking juice was investigated. VCF, T, and ΔP were evaluated in the ranges of 1.03–4.25, 20–60 °C, and 2–5 bar, respectively. According to the results illustrated in Figure 7a–c and Figure 8a–c, it is clear that the maximum protein removal (77.39%) and the highest permeate flux (302.76 L/h·m^2^) were obtained at the optimal conditions of VCF = 1.49, T = 60 °C, and ΔP = 5 bar by applying the RSM model. 

UF experiments were carried out at optimized conditions of treatment: VCF = 1.49, T = 60 °C, and ΔP = 5 bar. Table 5 represents the variation of the protein, COD, and salt contents of the tuna cooking juices, before and after the ultrafiltration process. According to the results, an essential removal of COD (93%) and protein (80%) was observed. However, the retention of salinity did not exceed 17%. These results were close to the optimized responses obtained using the BBD method.

### 3.5. Global Mass Balance 

The global mass balance in protein retention over the entire duration of the concentration of tuna cooking juice using the ultrafiltration process can be expressed as follows:

(14) $$C_{i}V_{i}=C_{p}V_{p}+C_{C}V_{C}$$

where: *V*_i_, *V_p_*, and *V_c_* are respectively the initial, permeate, and retentate volumes, and *C_i_*, *C_p_*, and *C_c_* are the initial, permeate, and retentate protein concentrations. Table 6 shows that the mass balance was satisfied for the protein removal when the VCF did not exceed 1.54 (an error of about 1%). It is not easy to obtain sharp precision in mass balance on filtration at high VCF (from VCF = 2.6) when the error is higher than 28%. At the end of the experiment, the main part of the dead volume can be recovered, but a small volume of concentrate always remains in pipes. Thus, there is no reason the masses should be exactly balanced.

### 3.6. Application of Polarization Concentration Model 

Table 7 illustrates the permeate flux and protein concentration values in the concentrate for each VCF value.

**Figure 9:**
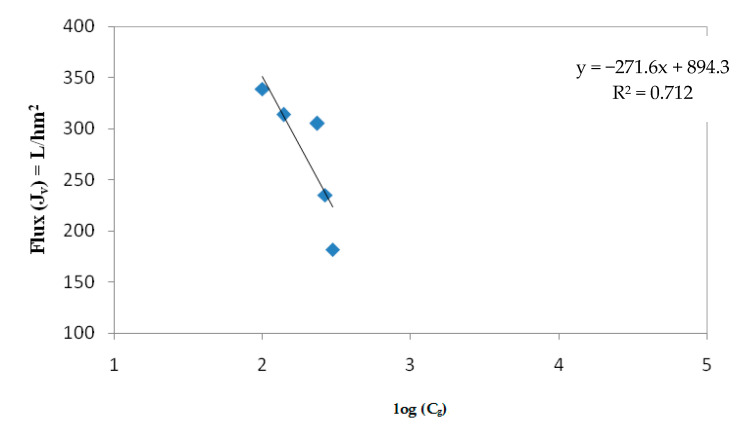
Variation of the permeate flux with log (*C_g_*).

The curve representing *J_v_* as a function of Ln *C_o_* (Figure 9) corresponds to a straight line from which the material transfer coefficient through the membrane (K = 271.6 L/h·m^2^) and the gel concentration (*C_g_* = 26.84 g/L) was determined.

Therefore, the solute concentration at which the flux becomes zero is almost *C_g_* = 26.84 g/L. This result confirms the error of 28.25% previously found indetermining the mass balance (Equation (14)).

### 3.7. Antifouling and Cleaning Study

**Figure 10:**
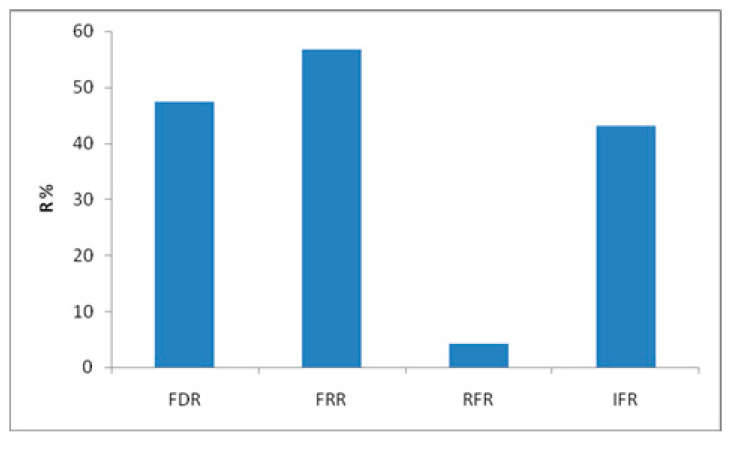
Permeate flux during filtration experiments with a ceramic UF membrane.

The membrane becomes less effective at separating components from the feed solutions during filtration. This fouling is coupled with partial deterioration of the membrane surface [30]. An anti-fouling study of the ceramic UF membrane used for protein removal was evaluated using four fouling parameters: FRR, FDR, RFR, and IFR. As depicted in Figure 10, the permeate flux of the membrane recovered to 56.85% (FRR) of the initial flux, and the FDR had a value of 48%. The decrease of the performance, in terms of permeate flux, corresponds to the accumulation and adsorption of molecules onto the membrane surface, leading to fouling. In addition, the low value of RFR (4.36%) shows that the removal of the adsorbed particles from the membrane surface can be achieved by simple hydraulic washing. However, the pore blockage is irreversible and can be quantified by the IFR measurement of 43.37%. This flux percentage can be recovered only by chemical cleaning [31]. From this finding, the attractive antifouling parameters prove that the ceramic UF membrane requiresa chemical treatment.

**Figure 11:**
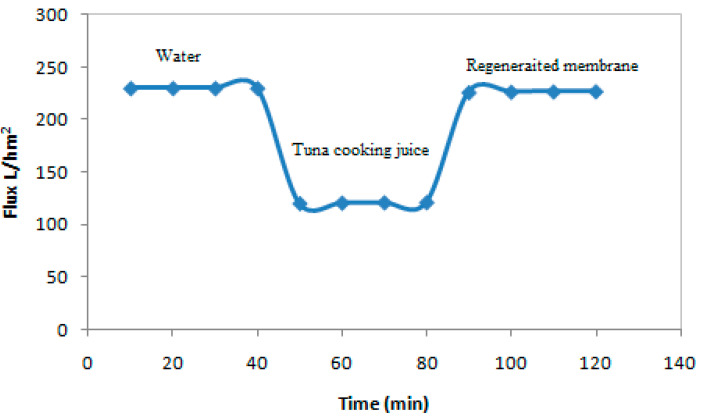
Recovery of membrane performance after chemical cleaning.

Figure 11 illustrates the water permeate flux before and after regeneration at 20 °C. The correction of the permeate flux during the treatment of tuna cooking juice at the temperature 20 °C was calculated from the following (Equation (15)) [31,32,33,34].

(15) $$J(20)=J(T)e^{\left(-0.0239(T-20)\right)}$$

where *T* is the temperature of the permeate flux, and *J*(20) and *J*(*T*) are the permeate flux at temperature 20 °C and at temperature *T*, respectively.

From Figure 11, the permeate flux during cooking juice treatment is less than the water permeate flux, due to pore blocking and the formation of a polarization layer on the membrane surface. Despite this, a significant restoration in the rate of water permeate flux was observed after chemical cleaning. The results demonstrated that the water permeability values were very close, once again confirming the efficiency of the cleaning process.

## 4. Conclusions

In this study, optimization of industrial tuna cooking juice treatment for protein recovery and wastewater purification using the response surface methodology (RSM) was achieved. The results revealed that the BBD of RSM was effectively used during this investigation. The protein rejection and permeate flux were mainly affected by VCF, T, and ΔP. The optimized conditions were VCF = 1.5, T = 60 °C, and TMP = 5 bar. Under these optimal conditions, 78% of protein removal and 302 L/h·m^2^ of permeate flux were achieved.

Regardless of membrane fouling, the polarization concentration and gel filtration model was successfully applied. Protein mass transfer coefficient and gel concentration were found to be K = 271.6 L/h·m^2^ and *C_g_* = 26.84 g/L, respectively. Chemical cleaning allow edentirely restoring the initial water permeability in this study.

Finally, based on the results found in this work, it is well understood that membrane separation technology has broad prospects for the advanced treatment and upgrading of tuna cooking juice. Water purification on a large scale using NF or RO can be applied after the UF, to enhance the water treatment quality by removing the residual pollutants and salts. This will help to obtain a water quality that meets the water standards for cooking juice, including salts with NF or drinking water standards with RO.

## Figures and Tables

**Table 1:** Physicochemical characteristics of the industrial tuna cooking juices.

Parameters	Unity	Sample 1	Sample 2	Sample 3
**pH**	-	7.14 ± 0.2	8.12 ± 0.2	6.9 ± 0.2
**Conductivity**	mS/cm	53.6 ± 0.4	52.8 ± 0.4	49.6 ± 0.4
**Turbidity**	NTU	1525 ± 100	1266 ± 100	1269 ± 100
**Protein**	g/L	6.32 ± 1	7.28 ± 1	8.4 ± 1

**Table 2:** Variables and levels in the Box–Behnken experimental design.

	Variables	Factor Levels		
		−1	0	1
Input factors				
VCF	X_1_	1.03	2.6	4.25
T (°C)	X_2_	20	40	60
ΔP (bar)	X_3_	2	3.5	7

**Table 3:** Box–Behnken experimental design and responses.

Run	Input Factors			Responses	
	VCF	T (°C)	ΔP (bar)	R Protein (%)	Permeate Flux (L/h·m²)
1	4.25	20	3.5	27	59
2	2.64	60	2	78	236
3	2.64	60	5	75	305
4	2.64	20	5	62	115
5	4.25	40	5	40	67
6	4.25	40	2	37	62
7	1.03	20	3.5	80	96
8	1.03	40	5	85	190
9	2.64	40	3.5	80	164
10	1.03	60	3.5	71	240
11	4.25	60	3.5	39	70.4
12	2.64	20	2	69	82
13	1.03	40	2	87	180

**Table 4:** Estimated coefficients for responses protein and permeate flux.

	b_0_	b_1_	b_2_	b_3_	b_12_	b_13_	b_23_	b_11_	b_22_	b_33_
**Protein**	80	**−22.5**	3.125	−1.125	5.25	1.25	1	**−17.25**	−8.5	−0.5
***p*** **-values**		**0.001**	0.1705	0.5642	0.1228	0.6467	0.7119	**0.0131**	0.0797	0.8877
**Flux**	170.914	**−55.95**	**62.425**	14.625	−33.15	−1.25	9	−56.2929		11.8571
***p*** **-values**		**0.0135**	**0.0092**	0.3319	0.1518	0.95	0.6563	0.0659		0.6247

**Table 5:** Characterization of tuna cooking juice before and after ultrafiltration process.

	COD (mg/L)	Salinity (g/L)	Protein (g/L)
Before ultrafiltration (Raw juice cooking)	24,250	37.4	6.32
After ultrafiltration	1750 (93%)	31 (17%)	1.3 (80%)

**Table 6:** The mass balance for the protein removal.

VCF	*C_i_V_i_* (g/L)	*C_p_V_p_* + *C_c_V_c_*	Error (%)
**1.03**	123.76	122.67	1.09
**1.54**	123.76	122.76	1.26
**2.6**	123.76	95.51	28.25
**3.77**	123.76	88.35	33.47
**4.26**	123.76	87.5	36.26

**Table 7:** Flux and protein concentration values in the concentrate for each VCF value.

VCF	*C_c_* (g/L)	Log *C_c_*	Flux (L/h·m^2^)
**1.03**	7.4	2	339
**1.54**	8.5	2.14	314
**2.6**	10.73	2.37	305
**3.77**	11.3	2.42	235
**4.26**	11.8	2.47	182

## Data Availability

Not applicable.
